# The Influence of Socioeconomic Factors on Health Parameters in Overweight and Obese Adults

**DOI:** 10.1371/journal.pone.0065407

**Published:** 2013-06-05

**Authors:** Nathalie T. Burkert, Éva Rásky, Franziska Großschädl, Johanna Muckenhuber, Wolfgang Freidl

**Affiliations:** Institute of Social Medicine and Epidemiology, Medical University Graz, Graz, Styria, Austria; The University of Texas M. D. Anderson Cancer Center, United States of America

## Abstract

The prevalence of being overweight and of obesity is increasing worldwide, and is associated with a high risk to health. Therefore, the aim of our study was to investigate whether normal weight, overweight and obese subjects of low, middle or high socioeconomic status (SES) differ with regard to their health behavior, health, quality of life, and the use of medical care. Data from the Austrian Health Interview Survey (ATHIS) 2006/07, comprising 3 groups of 1,077 individuals, each of whom were normal weight, overweight, or obese, respectively, and matched according to their age, sex and SES, were analyzed concerning health outcomes. The results show that subjects with a low SES differ significantly from those of high SES in terms of their health behavior, self-perceived health, levels of impairment, chronic conditions, quality of life, and health care. Additionally, obesity in adults is associated with sub-optimal dietary practices and worse health, poorer quality of life and medical care than normal weight and overweight individuals. A significant interaction between the weight class and SES was found concerning physical exercise, impairment due to health problems and chronic diseases. A low SES has a strong negative impact on health, especially in obese individuals. Therefore a continuous target group-oriented, non-discriminatory public health program is required, prioritizing obese subjects with low SES.

## Introduction

In the last two decades obesity has more than tripled in Europe, and has meanwhile become a serious public health problem, causing more than 1 million deaths and 12 million life years of illness each year [1]. Obesity is associated with increased medical costs [2], premature death, less healthy lifestyle choices, psychological problems as well as poor quality of life [1], [3]. An inverse relationship between educational level and body weight is well established [4]–[7]. Moreover, likelihood of becoming obese increases with lower income [8] and is inversely associated with the socioeconomic status (SES) [9], [10], even after adjusting for age [3], [11].

Health is affected by various factors as well as by their interaction. The SES, weight class as well as lifestyle factors all have an influence [12], [13]. Lifestyle factors associated with a health risk include tobacco smoking, alcohol consumption, sub-optimal dietary habits, and physical inactivity [1]. All these risk factors are more present in subjects with a low SES [1], [3], [14]. Smoking, an important contributor to health inequalities, is more common among obese individuals from a low social background [10]. Compared to smoking, the health risk attached to alcohol consumption is more complex. While moderate alcohol drinking leads to a lower mortality rate [15], both abstinence and excessive consumption are detrimental to health [16]. Both a low SES and obesity are related to unhealthy food consumption [17]. Additionally, the SES has a stronger impact on physical activity amongst obese women and men compared to normal weight subjects [18].

There is evidence for the existence of a social gradient in health: when compared to people of high SES, people of low SES show an increased prevalence of morbidity [16], a high level of health complaints [19], and have a lower life expectancy by 7 years [20]. Obese individuals with a low SES self-report worse self-perceived health and quality of life [10], [18], [21], [22]. Moreover, the influence of the SES on these variables is greater in obese compared to normal weight subjects [18], [21]. Nevertheless current clinical guidelines and public health statements often link the Body Mass Index (BMI) to diseases, regardless of the individual’s situation (e.g. age, SES, genes) [23].

In conclusion, it can be stated that a low SES *per se* is associated with a higher health risk and is a risk factor for morbidity and mortality [16], [23]. Although the highest prevalence of obesity is found in the low-educated and the lowest prevalence in the highest educational bracket, obesity has meanwhile increased in all SES groups equally [24]. Studies which analyze the impact of socioeconomic factors across different weight classes are scarce. Therefore, the aim of this study is to analyze differences in health-related behavior, health, medical treatment, and quality of life of obese, overweight and normal weight subjects of either high, middle or low SES.

## Methods

### Study Population

The sample used for this study was taken from the Austrian Health Interview Survey (AT-HIS) 2006/07 [25]. The AT-HIS is a standardized survey which is conducted at regular intervals in Austria (currently every eight years). The subjects included are a representative sample of the Austrian population. They are chosen from the central population register and are distributed across Austria’s different geographic regions. The AT-HIS is part of the European Health Interview Survey (E-HIS; http://www.euhsid.org), which is an important survey of high quality.

Overall, 15,474 participants were interviewed aged 15 years and older (54.7% female; response rate: 63.1%). The study was conducted through home-based personal interviews with interviewees representative of the Austrian population. Analyses were then carried out on the basis of a sample population comprising normal weight, overweight, and obese subjects aged 20 years and older, with each of these three weight classes having matching profiles in terms of the ages, genders and SES of the subjects within each weight class (N = 3 231).

### Matching Process

Overall, 13,345 subjects (53.4% female) who were 20 years and older participated in the study. 244 of these individuals were underweight, 6,297 were normal weight, 4,994 were overweight, and 1,810 were obese.

As a first step, subjects who were obese were identified (N = 1 810). All obese subjects were categorized according to their sex, age (in age-groups spanning 5 years, e.g., 20- to 24-year-olds), and socioeconomic status (low (SES≤6), middle (SES = 7,8) or high (SES≥9) SES, based on percentiles 33.33 and 66.67), with each such obese subject then matched with one normal weight individual and one overweight individual of the same sex and SES, and in the same age-group. 59.5% of all obese subjects could be included in the analyses, since not all obese individuals could be matched with normal weight or overweight subjects of the same sex, age, and SES. Therefore, the total number of subjects analyzed, was 3,231 (47.4% female; 24.2% of all subjects who were 20 years and older, and participated in the AT-HIS; comprising 1,077 individuals with obesity, 1,077 individuals of normal weight and 1,077 overweight individuals). Descriptive information about the subjects who were included in the analyses is shown in Table 1.

**Table 1:** Data definition and structure for normal weight, overweight and obese subjects, respectively, in each SES group.

Age	Sex	
	m	w
**20–24**	8	5
**25–29**	8	8
**30–34**	10	8
**35–39**	13	16
**40–44**	13	20
**45–49**	25	21
**50–54**	19	13
**55–59**	33	28
**60–64**	23	20
**65–69**	19	12
**70–74**	9	9
**75–79**	7	4
**80–84**	2	4
**85+**	0	2
**Total**	189	170

*Note.* Data source: Austrian Health Interview Survey (ATHIS) 2006/07. Number of subjects. Analyses were calculated with subjects matched by age, sex, and socio-economic status (N = 3231). The subjects represented in this study were included nine times respectively: normal weight subjects with a low/middle/high SES, overweight subjects with a low/middle/high SES, and obese subjects with a low/middle/high SES.

### Ethical Approval

The study was carried out in compliance with the declaration of Helsinki. The ethics committee of the Medical University of Graz approved this study (EK-number: 24–288 ex 11/12).

### Measurements

Face-to-face interviews were conducted, questioning the subjects about their socio-demographic characteristics, diseases, health-related behavior, and psychological issues.

The independent variables in this study were the weight class (normal weight: BMI ≥18.5 kg/m^2^ and <25 kg/m^2^, overweight: BMI ≥25 kg/m^2^ and <30 kg/m^2^ or obesity: BMI ≥30 kg/m^2^) and the SES (low (SES≤6), middle (SES = 7,8) or high (SES≥9), based on percentiles 33.33 and 66.67). The SES was calculated using the following variables: net equivalent income, level of education, and occupation. Net equivalent income was calculated based on an equivalence scale provided by the OECD [26], and divided based on quintiles. Level of education was measured by an ordinal variable distinguishing between basic education (up to 15 years of age), apprenticeship/vocational school, secondary education without diploma, secondary education with diploma, and university. The occupation of the subjects was also differentiated into five different levels: (1) unskilled worker, (2) apprentice/skilled worker, (3) self-employed/middle job, (4) qualified job/academic, (5) executive position. To verify the combination of factors that served to calculate the SES, correlations with the different factors were calculated. They ranged between r = .70 and r = .80.

The dependent variables concerning health-related behavior were smoking habits (cigarettes per day), frequency of alcohol consumption during the last 28 days (expressed in terms of the number of days on which alcohol was consumed), dietary habits (1 = vegetarian avoiding all animal products, 2 = vegetarian consuming milk/eggs, 3 = vegetarian consuming fish and/or milk/eggs, 4 = meat diet combined with lots of fruit and vegetables, 5 = meat diet with little meat, 6 = meat diet with a lot of meat), and the physical exercise score (total MET score) [27]. Physical exercise was measured using the short version of the International Physical Activity Questionnaire (IPAQ), a self-reported instrument which asks for an estimate of total weekly physical activity (walking/vigorous and moderate-intensity activity) during the previous week. The short version of the IPAQ does not discriminate between leisure and non-leisure physical activity. The total MET score was calculated by weighting the minutes reported for each activity per week using a MET energy expenditure estimate that was assigned to each category [27].

The dependent variable concerning quality of life was measured using the short version of the WHOQOL (WHOQOL-BREF) [28]. The four domain scores (physical health, psychological health, social relationships, and environment) were calculated. Domains ranged between a score of 4 and 20.

The dependent variables focusing on ill-health included self-perceived health, ranging from 1 (very good) to 5 (very bad), and health impairment, ranging from 1 (highly impaired) to 3 (not impaired). The presence of 17 chronic conditions (hypertension, apoplectic stroke, cardiac infarction, cancer, gastric or intestinal ulcer, diabetes, arthritis, osteoporosis, bronchitis, tinnitus, cataract, allergies, asthma, urinary incontinence, mental illness (anxiety disorder or depression), migraine, and other chronic condition) was assessed. Each condition was coded as present (1) or absent (0). The total frequency score was calculated by summing up the chronic conditions identified (0–17, sum index). Additionally, a vascular risk score was calculated by summing up the variables “hypertension”, “enhanced blood cholesterol level”, “diabetes”, and “smoking” (0–4, sum index). Each variable was coded as present (1) or absent (0).

A dependent variable concerning medical care was created as the sum index of the number of doctors consulted in the last 12 months (0–8, sum index). Each of the 8 medical treatments (e.g. general practitioner, specialist) was coded as “consulted” (1) or “not consulted” (0). The number of vaccinations (e.g. influenza, hepatitis) was also analyzed by calculating a sum index concerning 8 different vaccinations (0–8, sum index), coded as present (1) or absent (0). Furthermore preventive medical care was analyzed by calculating a sum index of “preventive check-ups”, “mammography”, “check-up prostate gland”, and “Papanicolaou test” (0–4, sum index). Each variable was coded on the basis of whether a subject had ever employed the service (1) or not (0).

### Statistical Analyses

As a first step, normal weight subjects, overweight subjects, and obese subjects were matched according to their sex and age (to eliminate their influence on the dependent variables), and socioeconomic status (low, middle, or high based on percentiles 33.33 and 66.67). As a result we carried out our analysis using a 3×3 design based on subjects with a different weight class and various SES (since these were the key variables we wanted to analyze), but with each of the three weight classes having the same profile in terms of the ages and genders of the subjects within each class. After matching, the total number of subjects analyzed was 3 231 (equating to N = 359 for each SES group within each weight class; see Table 1).

In order to analyze the differences and variations between individuals from the different weight classes and SES, in terms of their health behavior, health, quality of life, and medical care, multivariate analyses of variance were conducted for each domain. Tukey HSD was used as a post-test. For all analyses *p*-values <*0.05* were considered statistically significant. All analyses were calculated using SPSS software (version 20.0).

## Results

### Health Behavior

Our multivariate analyses of variance have shown a significant main effect for both the weight class (p = .000) and SES (p = .000). Overall, obese subjects and those of a low SES demonstrate poorer health behavior. Obese subjects differ significantly from normal weight and overweight persons in terms of their dietary habits insofar as obese subjects have a higher meat intake (p = .000). No difference was found between the weight groups in relation to their levels of physical exercise (p = .604) or alcohol consumption (p = .223), nor in relation to their smoking behavior (p = 916).

Subjects with a low SES smoke significantly more (p = .000), drink less alcohol (p = .000), and are more physically active (p = .001) than those with a middle and high SES. No difference regarding dietary habits was found between the SES groups (p = .133).

**Figure 1:**
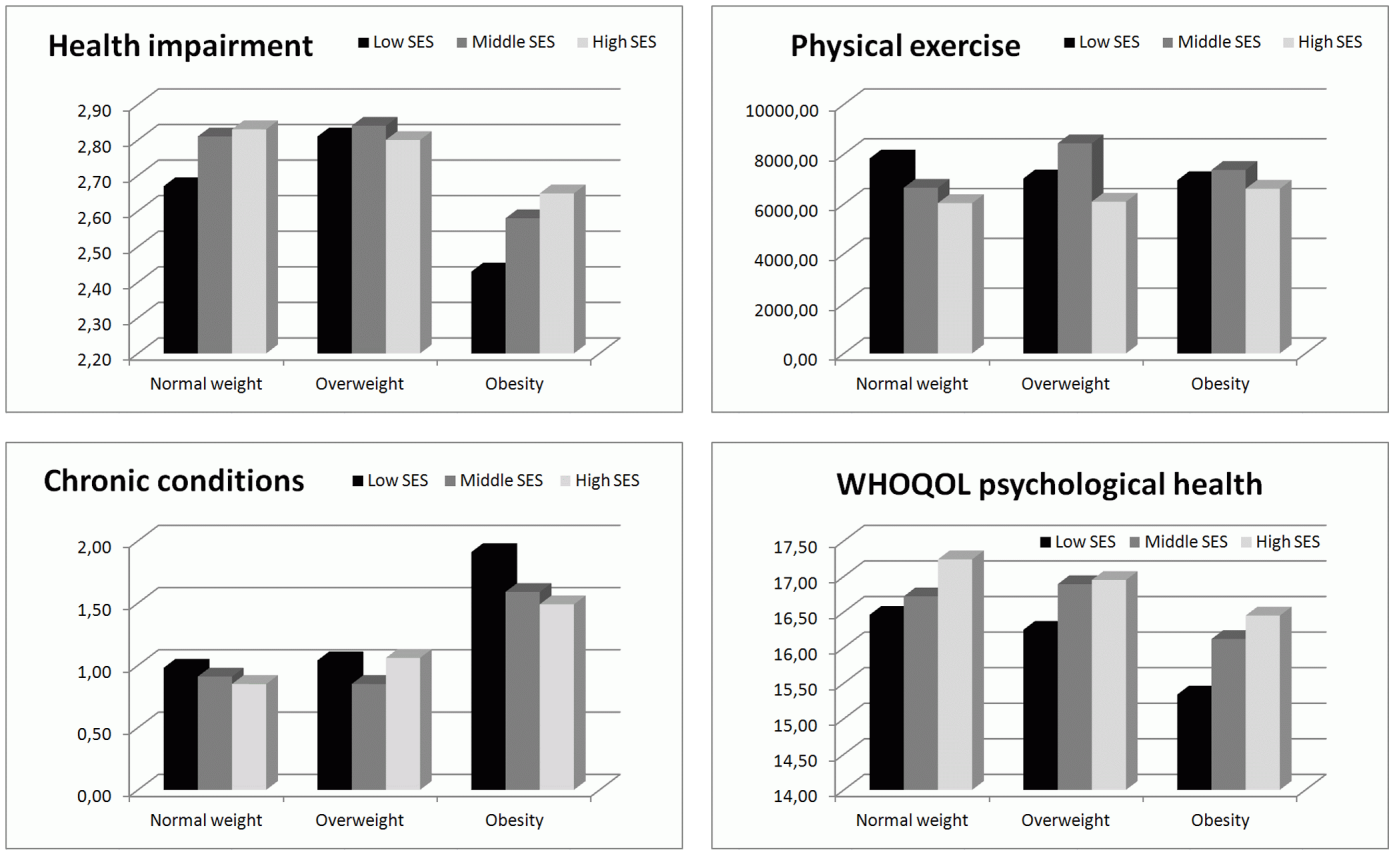
Impairment due to health problems, number of chronic conditions, physical exercise, and quality of life regarding psychological health in normal weight, overweight and obese subjects with a different SES. *Note.* Data source: Austrian Health Interview Survey (ATHIS) 2006/07. Analyses were calculated with subjects matched by age, sex, and SES (N = 3231). Health impairment, physical exercise, and WHOQOL (Quality of life) regarding psychological health: higher score means better results; chronic conditions: higher score means worse results.

An interaction between weight class and SES was significant for physical exercise (p = .021). Figure 1 shows that the SES has a different impact on physical exercise according to whether the subjects are normal weight, overweight or obese. In normal weight subjects, physical exercise declines with rising SES, whereas in overweight and obese individuals those with a middle SES perform more physical exercise than subjects with a low or high SES. All results are shown in Table 2.

**Table 2:** Health behavior in normal weight, overweight and obesity.

	Low SES		Middle SES		High SES		Test Statistics		
	M	SD	M	SD	M	SD	weight class	SES	weight class*SES
**Health behavior**							**.000**	**.000**	.501
**Normal weight**									
Physical exercise (total MET score)^1^	7829.24	8151.76	6652.57	7188.48	6034.87	6800.60	.604	**.001**	**.021**
Number of cigarettes per day^2^	5.51	9.32	4.24	8.86	2.56	6.40	.916	**.000**	.784
Alcohol consumption (number of days within 4 weeks)^2^	5.66	8.33	6.60	8.79	7.26	8.52	.223	**.000**	.958
Dietary habits^3^	4.94	0.80	4.92	0.81	4.84	0.77	**.000**	.133	.883
**Overweight**									
Physical exercise (total MET score)	7026.58	8206.27	8435.20	8646.18	6094.54	6995.96			
Number of cigarettes per day	5.24	9.69	3.58	7.80	3.04	6.97			
Alcohol consumption (number of days within 4 weeks)	5.92	8.30	6.88	8.99	7.85	8.79			
Dietary habits	5.06	0.75	5.02	0.77	5.01	0.79			
**Obesity**									
Physical exercise (total MET score)	6953.98	8713.87	7369.27	8892.35	6613.38	8610.54			
Number of cigarettes per day	5.29	10.42	4.04	8.99	2.81	7.24			
Alcohol consumption (number of days within 4 weeks)	5.64	8.66	6.20	8.68	6.88	8.60			
Dietary habits	5.15	0.77	5.10	0.79	5.10	0.80			

*Note.* Data source: Austrian Health Interview Survey (ATHIS) 2006/07. M = mean, SD = standard deviation.

1 higher score means better results,

2 higher score means worse results,

3 higher score means more meat consumption. Analyses were calculated with subjects matched by age, sex, and socio-economic status (N = 3231).

### Health

In the domain of health, the multivariate analysis of variance showed a significant main effect for both the weight class (p = .000) and SES (p = .000). Overall, subjects in a higher weight class or with a lower SES are in a worse state of health. The interaction SES*weight class (p = .014) was also statistically significant and implies that the SES has a greater impact in the case of obese subjects than in the case of overweight and normal weight subjects. Obese subjects report that they are generally in a poorer state of health (p = .000), are suffering from more impairment due to disorders (p = .000), and are suffering from more chronic diseases (p = .000). Moreover, the vascular risk is increased in obese compared to normal weight, and overweight persons (p = .000).

We found a significant main effect for the SES for all health variables. Subjects with a high SES self-report better health (p = .000), less impairment (p = .000), and suffer from significantly less chronic diseases (p = .003). Additionally, the vascular risk is lower for subjects with a high SES (p = .042).

We found a significant weight class*SES interaction in relation to impairment due to disorders (p = .003), as well as in relation to the number of chronic conditions (p = .037). Figure 1 shows that in normal weight, overweight and obese subjects the SES has a different impact on impairment due to health problems. Normal weight subjects of low SES report more impairment than normal weight individuals with a middle or high SES. Amongst overweight individuals those with a middle SES report the least impairment due to health problems. The difference in the level of health impairment, when comparing individuals of lower and higher SES, is greater for obese subjects than it is for normal weight and overweight subjects. The number of chronic conditions declines with rising SES in normal weight subjects. In overweight individuals those with a middle SES report the least number of chronic conditions. Again, the difference in the number of chronic conditions when comparing individuals of lower and higher SES is greater for obese subjects than it is for normal weight and overweight subjects. All results are shown in Table 3.

**Table 3:** Health in normal weight, overweight and obesity.

	Low SES		Middle SES		High SES		Test Statistics		
	M	SD	M	SD	M	SD	weight class	SES	weight class*SES
**Health**							**.000**	**.000**	**.014**
**Normal weight**									
Self-reported health generally^2^	1.68	0.82	1.52	0.78	1.36	0.69	**.000**	**.000**	.149
Impairment^1^	2.67	0.60	2.81	0.49	2.83	0.42	**.000**	**.000**	**.002**
Chronic conditions^2^	0.98	1.40	0.91	1.28	0.85	1.15	**.000**	**.003**	**.037**
Vascular risk^2^	1.95	0.75	2.03	0.70	2.09	0.65	**.000**	**.042**	.235
**Overweight**									
Self-reported health generally	1.72	0.78	1.52	0.78	1.36	0.69			
Impairment	2.81	0.49	2.84	0.45	2.80	0.48			
Chronic conditions	1.04	1.39	0.85	1.24	1.06	1.42			
Vascular risk	2.06	0.79	2.11	0.71	2.21	0.81			
**Obesity**									
Self-reported health generally	2.34	0.98	2.04	0.79	1.90	0.78			
Impairment	2.43	0.75	2.58	0.61	2.65	0.58			
Chronic conditions	1.91	1.97	1.59	1.54	1.49	1.58			
Vascular risk	2.48	1.02	2.45	0.92	2.45	0.95			

*Note.* Data source: Austrian Health Interview Survey (ATHIS) 2006/07. M = mean, SD = standard deviation.

1 higher score means better results,

2 higher score means worse results. Analyses were calculated with subjects matched by age, sex, and socio-economic status (N = 3231).

### Quality of Life

Regarding the quality of life, the main effect for the weight class (p = .000), SES (p = .000), and also their interaction (weight class*SES; p = .000) showed a statistically significant effect. Overall, obese subjects and those with a lower SES have a worse quality of life. Moreover, the SES has a greater impact in the case of obese subjects than in the case of normal weight and overweight subjects. Obese subjects have the worst quality of life in the domains physical health (p = .000), psychological health (p = .000), social relationships (p = .000), and also environment (p = .000).

Moreover, subjects with a low SES have the lowest quality of life in all four domains (p = .000 for all subtests).

Although the interaction for the combination of the four domains of the WHOQOL turned out to be significant, showing that, overall, the SES has a greater impact for obese subjects than for normal weight and overweight subjects, no statistically significant difference was found across the various domains regarding quality of life. However, the subtest “psychological health” revealed a tendency according to which the SES has more impact in subjects with obesity (p = .069). Figure 1 shows that in normal weight subjects, quality of life rises on a nearly linear basis with rising SES, whereas in overweight individuals a big gap exists between subjects with a low SES and those with a middle or high SES. In individuals with obesity the difference in quality of life between subjects of low SES and those with a middle and high SES is the greatest. All results are presented in Table 4.

**Table 4:** Quality of life in normal weight, overweight and obesity.

	Low SES		Middle SES		High SES		Test Statistics		
	M	SD	M	SD	M	SD	weight class	SES	weight class*SES
**Quality of life**							**.000**	**.000**	**.000**
**Normal weight**									
WHOQOL physical health^1^	17.12	2.74	17.70	2.55	18.12	2.13	**.000**	**.000**	.123
WHOQOL psychological health^1^	16.46	2.28	16.72	2.28	17.24	1.87	**.000**	**.000**	.069
WHOQOL social relationships^1^	16.48	2.58	16.76	2.57	17.13	2.25	**.000**	**.000**	.354
WHOQOL environment^1^	16.12	2.14	16.57	1.91	17.18	1.65	**.000**	**.000**	.633
**Overweight**									
WHOQOL physical health	17.03	2.73	17.70	2.13	17.75	2.29			
WHOQOL psychological health	16.25	2.29	16.89	1.93	16.95	2.01			
WHOQOL social relationships	16.40	2.50	16.76	2.57	17.13	2.25			
WHOQOL environment	15.92	2.17	16.54	1.85	16.84	1.84			
**Obesity**									
WHOQOL physical health	15.55	3.37	16.34	2.74	16.93	2.58			
WHOQOL psychological health	15.34	2.69	16.12	2.05	16.45	2.05			
WHOQOL social relationships	15.81	2.85	16.48	2.56	16.65	2.22			
WHOQOL environment	15.39	2.42	16.03	1.95	16.45	1.92			

*Note.* Data source: Austrian Health Interview Survey (ATHIS) 2006/07. M = mean, SD = standard deviation.

1 higher score means better results. Analyses were calculated with subjects matched by age, sex, and socio-economic status (N = 3231).

### Health Care

Our multivariate analyses for medical care have shown a significant main effect for both the weight class (p = .000) and SES (p = .000), showing that, overall, obese subjects and those of a low SES demonstrate worse health care practices.

Obese subjects need more medical treatment (p = .000) and are vaccinated less often (p = .004). No difference was found regarding preventive check-ups (p = .145) between the subjects with a different weight class.

Individuals with a high SES consult doctors significantly more often (p = .004), are vaccinated more often (p = 000), and additionally make more frequent use of preventive care (p = .000) than individuals with a low SES.

The weight class*SES interaction was not significant for health care (p = .492). All results are reported in Table 5.

**Table 5:** Health care in normal weight, overweight and obesity.

	Low SES		Middle SES		High SES		Test Statistics		
	M	SD	M	SD	M	SD	weight class	SES	weight class*SES
**Health care**							**.000**	**.000**	.492
**Normal weight**									
Medical treatment (e.g. general practitioner, specialist)^1^	1.57	1.24	1.59	1.22	1.71	1.43	**.000**	**.004**	.689
Vaccinations^1^	2.80	2.11	3.17	2.09	3.52	2.20	**.004**	**.000**	.199
Preventive medical care^1^	1.42	1.07	1.65	1.02	1.63	1.01	.145	**.000**	.519
**Overweight**									
Medical treatment (e.g. general practitioner, specialist)	1.43	1.13	1.48	1.20	1.70	1.26			
Vaccinations	2.64	3.17	2.95	2.04	3.63	2.07			
Preventive medical care	1.40	1.08	1.52	1.05	1.66	1.00			
**Obesity**									
Medical treatment (e.g. general practitioner, specialist)	1.77	1.24	1.85	1.34	1.89	1.30			
Vaccinations	2.28	1.98	2.91	1.94	3.44	2.05			
Preventive medical care	1.37	1.06	1.47	1.02	1.60	0.98			

*Note.* Data source: Austrian Health Interview Survey (ATHIS) 2006/07. M = mean, SD = standard deviation.

1 higher score means better results. Analyses were calculated with subjects matched by age, sex, and socio-economic status (N = 3231).

## Discussion

Overall, our results show obese subjects as well as persons with a low SES to be in the poorest state of health: they self-report poorer health, more impairment due to disorders, and more chronic diseases. Moreover, vascular risk is significantly increased and the quality of life significantly reduced for obese subjects and persons of low SES. This is in line with previous studies revealing that obesity is associated with premature death, psychological problems as well as poor quality of life [1], [3], [18], [22], and that people with a low SES show worse self-reported health [13], a high level of health complaints [19], a higher prevalence of morbidity and mortality [16] and unfavorable psychosocial factors [17]. Interestingly, overweight subjects do not differ from normal weight persons regarding health or quality of life. Nevertheless, there seems to be a gradient associated with a higher weight class regarding these variables, showing that health and quality of life in overweight persons are better than in obese individuals and worse than in normal weight individuals. However, the difference from normal weight persons is not statistically significant.

Most importantly, our findings show that the impact of the social background on health is higher for obese subjects than for normal weight and overweight subjects. In obese persons the social background has the greatest influence on the amount of physical exercise, impairment due to disorders, and the number of chronic conditions. Studies report that the impact of obesity on self-perceived health is greater among women of low educational level [21] or low SES [18]. Since our findings also show that the number of chronic diseases is increased in obese persons of low SES, they emphasize the influence of socioeconomic factors on the health of obese individuals. Additionally we were able to show that the SES has a greater impact on the overall quality of life of obese persons.

The dietary habits of obese persons differ from normal weight and overweight subjects insofar as obese individuals have a higher meat intake. Concerning dietary habits we tried to generate a variable that would reflect the amount of animal fat intake. Our results showed that the amount of animal fat intake is increased in obese subjects.

No statistically significant difference was found regarding the level of physical exercise, smoking habits or alcohol consumption between the different weight groups. Nevertheless, our results showed that subjects with a low SES demonstrate worse health behavior. They smoke more cigarettes per day and are less physically active. This corresponds with the results of studies showing smoking to be more common among subjects with a low SES [16], and people of a lower educational level or with a lower income [22]. Wee et al. [29] have found that the deficiency in physical activities is greater for women in low social positions. Results of previous studies have shown men with a lower SES to be more physically active at work, but less so during leisure time [30], and that obesity is related to lower levels of non-leisure physical activities [31]. We were unable to verify this since the total MET score [27] fails to differentiate between physical activity at work or during leisure time. Hence, further studies are required to analyze physical activity in more detail.

In our study, people of high SES drink alcohol more often over the course of 28 days than those of low SES. The association between alcohol consumption and health is a complex factor when assessing health risk. While moderate alcohol intake is related to a lower mortality rate [15], both abstinence and excessive consumption seem to be detrimental to health [16]. Moreover, studies have shown that people of a low SES are more likely to be abstinent, but that when such individuals consume alcohol, they more often show problematic drinking patterns [20]. Since we only asked for the number of days on which alcohol was consumed during the last 28 days, no statement can be made as to the amount of alcohol that had been drunk. Perhaps subjects with a high SES are more likely to drink alcohol moderately, which goes along with reduced mortality rates [15]. Therefore the health risk linked to alcohol intake might also be higher for the low-SES group.

Additionally, our results evidence that obese subjects need more medical treatment, a phenomenon that might be due to their impaired health. Subjects with a high SES in our study seek medical treatment more often, are vaccinated against more diseases, and regularly undergo preventive check-ups. There is also evidence that predictors of medical screening include place of residence (urban vs. rural), marital status, education, SES, and the presence of one or more chronic conditions [32]–[34].

Among the strengths of our study is the representative nature of the sample population, matching according to age and sex across each weight class, as well as the standardized measurement of all variables. One potential limitation of our results is that the survey was based on cross-sectional data; therefore no statement can be made concerning morbidity and mortality. Hence, further longitudinal studies are required to substantiate our results. Further limitations include the measurement of dietary habits as a self-reported and not validated variable, and the fact that there was no attempt at differentiating between detailed eating habits, e.g. the amount of carbohydrates which individuals consume.

### Conclusions

In conclusion, our study has shown health to be impaired in obesity and subjects of low SES. Most importantly, our findings indicate that the impact of the SES on health is greater in obese subjects than in normal weight or overweight subjects. While a number of studies have analyzed differences in health, health behavior, or quality of life on the basis of the SES and/or weight class of subjects, we are not aware of any published study that surveyed all these variables together. The main implication of our findings is that in the future, socioeconomic factors should be taken into account when calculating the health risk. Moreover, a continuous target group-oriented, non-discriminatory public health program is required, prioritizing obese subjects of low SES.
